# RKIP: A Key Regulator in Tumor Metastasis Initiation and Resistance to Apoptosis: Therapeutic Targeting and Impact

**DOI:** 10.3390/cancers10090287

**Published:** 2018-08-24

**Authors:** Apostolos Zaravinos, Benjamin Bonavida, Ekaterini Chatzaki, Stavroula Baritaki

**Affiliations:** 1 Department of Life Sciences, School of Sciences, European University Cyprus, 2404 Nicosia, Cyprus; a.zaravinos@euc.ac.cy; 2 Centre for Risk and Decision Sciences (CERIDES), 2404 Nicosia, Cyprus; 3 Department of Microbiology, Immunology, and Molecular Genetics, David Geffen School of Medicine, University of California Los Angeles, Los Angeles, CA 90095, USA; bbonavida@mednet.ucla.edu; 4 Laboratory of Pharmacology, Medical School, Democritus University of Thrace, 68100 Alexandroupolis, Greece; achatzak@med.duth.gr; 5 Division of Surgical Oncology, School of Medicine, University of Crete, Heraklion, 71500 Crete, Greece

**Keywords:** RKIP, cancer, resistance, metastasis, EMT, therapy

## Abstract

RAF-kinase inhibitor protein (RKIP) is a well-established tumor suppressor that is frequently downregulated in a plethora of solid and hematological malignancies. RKIP exerts antimetastatic and pro-apoptotic properties in cancer cells, via modulation of signaling pathways and gene products involved in tumor survival and spread. Here we review the contribution of RKIP in the regulation of early metastatic steps such as epithelial–mesenchymal transition (EMT), migration, and invasion, as well as in tumor sensitivity to conventional therapeutics and immuno-mediated cytotoxicity. We further provide updated justification for targeting RKIP as a strategy to overcome tumor chemo/immuno-resistance and suppress metastasis, through the use of agents able to modulate RKIP expression in cancer cells.

## 1. Introduction

The RAF-kinase inhibitor protein [RKIP or Phosphatidylethanolamine-binding protein-1 (PEBP1)] has been critically involved in the regulation of distinct biological processes, through interactions with several signaling cascades, such as the mitogen-activated protein kinase (MAPK), nuclear factor kappa-light-chain-enhancer of activated B cells (NF-κB), G protein-coupled receptors (GPCR) and glycogen synthase kinase3β (GSK3β) pathways [1,2,3,4,5,6,7]. The reduction or loss of RKIP expression has been associated with the initiation and progression of multiple diseases, including cancer.

In numerous human cancers, RKIP was shown to act as an endogenous onco-suppressing protein affecting negatively tumor cell survival, proliferation, and metastasis [8]. The most well-reported role of RKIP is in the metastasis suppression [9,10,11,12,13,14,15]. Metastasis comprises of multiple steps, including the epithelial-to-mesenchymal transition (EMT), cellular migration, invasion, intravasation into blood or lymph vessels, extravasation from circulatory vessels, and tumor cell colonization at new tissue sites [16]. Each metastatic step encompasses intricate cell–cell interactions and signaling pathways. As a metastasis suppressor, RKIP impedes different stages of the aforementioned processes and its high level of expression is predictive of a better clinical outcome [9,14,17,18,19,20,21].

RKIP overexpression is further reported to reverse tumor chemo/immune/radio-resistance and support anticancer host immunosurveillance [22]. We and others have shown that cancer cells acquire therapeutic resistance through constitutive activation of multiple survival pathways that help them to evade apoptosis [23,24,25,26,27,28,29,30,31,32,33,34].

The main goal of this review is to update the vital impact of the RKIP expression levels in cancer cells on their sensitivity to cytotoxic therapies and on their metastatic potential. Accordingly, we mainly discuss the regulatory role of RKIP on multiple signaling modules reported so far to be involved in metastasis initiation and therapeutic resistance in various cancer types. We also discuss the most recent advances on the prognostic and therapeutic applications of RKIP expression levels in cancer.

## 2. RKIP Expression Patterns across Different Cancer Types

RKIP has been identified as an important protein in various cancer types, including those of prostate, melanoma, colorectal, liver, breast, urinary bladder, nasopharyngeal carcinoma, skin, lung and others [17,35,36,37,38,39,40,41,42,43,44,45,46,47]. Several studies have shown that RKIP exhibits low expression levels in various cancers and it is often absent in metastasis [8,9,17,19,43,45,48,49,50,51,52,53,54,55,56,57,58,59,60]. RKIP loss has been suggested to result by hypermethylation of its promoter [41].

**Figure 1:**
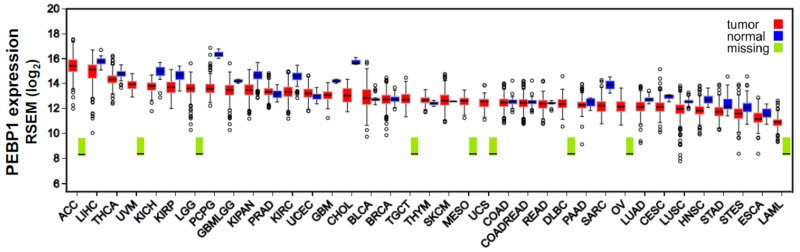
RKIP (PEBP1) expression across 37 TCGA cancers and their corresponding normal tissue. RKIP is downregulated in the majority of the different cancer types compared to the normal tissues. RNA-seq data quantification was performed using RSEM [61] following log_2_ transformation. Tumor sample number, ranges in the values of RKIP mRNA expression levels, and differential fold change between each tumor type and the corresponding normal tissue is presented in Table S1. Abbreviations: AAC, adrenocortical carcinoma; LIHC, liver hepatocellular carcinoma; THCA, thyroid carcinoma; UVM, uveal melanoma; KICH, kidney chromophobe carcinoma; KIRP, kidney renal papillary cell carcinoma; LGG, brain lower-grade glioma; PCPG, pheochromocytoma and paraganglioma; GBMLGG, glioma; KIPAN, Pan-kidney cohort (KICH+KIRC+KIRP); PRAD, prostate adenocarcinoma; KIRC, kidney renal clear cell carcinoma; UCEC, uterine corpus endometrial carcinoma; GBM, glioblastoma multiforme; CHOL, cholangiocarcinoma; BLCA, bladder urothelial carcinoma; BRCA, breast invasive carcinoma; TGCT, testicular germ cell tumors; THYM, thymoma; SKCM, skin cutaneous melanoma; MESO, mesothelioma; UCS, uterine carcinosarcoma; COAD, colon adenocarcinoma; COADREAD, colorectal adenocarcinoma; READ, rectum adenocarcinoma; DLBCL, diffuse large B-cell lymphoma; PAAD, pancreatic adenocarcinoma; SARC, sarcoma; OV, ovarian serous cystadenocarcinoma; LUAD, lung adenocarcinoma; CESC, cervical squamous cell carcinoma and endocervical adenocarcinoma; LUSC, lung squamous cell carcinoma; HNSC, head and neck squamous cell carcinoma; STAD, stomach adenocarcinoma; STES, stomach and esophageal carcinoma; ESCA, esophageal carcinoma; LAML, acute myeloid leukemia.

Here, for instance, we measured RKIP mRNA expression across 37 different cancer types, using data from The Cancer Genome Atlas (TCGA) platform (https://cancergenome.nih.gov/), corroborating its downregulation in the majority of them compared to the normal tissues. Our analysis shows that RKIP exhibits its highest levels in adrenocortical carcinoma (ACC), liver hepatocellular carcinoma (LIHC), and thyroid carcinoma (THCA), and its lowest expression was detected in acute myeloid leukemia (LAML), esophageal carcinoma (ESCA), and stomach and esophageal carcinomas (STES) (Figure 1).

## 3. RKIP Regulation

**Figure 2:**
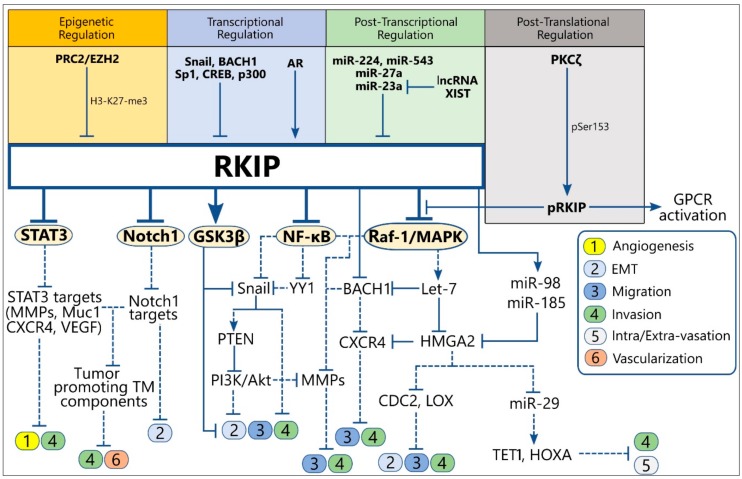
Schematic representation of the upstream regulators of RKIP expression (up) and the direct and indirect downstream RKIP targets involved in the regulation of metastasis initiation (down). Solid lines indicate the physiological function of each protein on the expression of downstream targets, while the dotted lines show the downstream effect(s) of the indicated protein after alteration of its levels by RKIP. Abbreviations: TM, Tumor microenvironment; EMT, Epithelial-to-Mesenchymal Transition; MMPs, Matrix Metalloproteinases; AR, Androgen Receptor; pRKIP, phosphorylated RKIP; PRC2, Polycomb Repressing Complex 2; EZH2, Enhancer of Zeste Homolog 2.

RKIP expression is regulated at multiple levels (Figure 2). At the epigenetic level, the RKIP promoter is frequently found methylated in most cancers. We have reported enhancer of zeste homolog 2 (EZH2)-mediated H3-K27-me3 and H3-K9-me3 motifs in the RKIP promoter of prostate and breast cancer cell lines [62]. We have also used the Spearman’s test to calculate the correlation between RKIP DNA methylation and mRNA expression profiles over 367 matched skin cutaneous melanoma samples, using “level 3” CpG site methylation and gene expression data extracted from the TCGA-SKCM dataset (https://portal.gdc.cancer.gov/projects/TCGA-SKCM). We chose skin melanoma for this analysis due to the high number of metastatic cases provided by the TCGA platform. The CpG methylation and gene expression data were paired using the gene's Entrez ID number (5037). We found that RKIP mRNA expression is negatively correlated with the corresponding PEBP1 methylation probe (cg00091483) in position 118574757 of chr12 (correlation coefficient = −0.412, *p* < 0.0001, q < 0.0001, mean expression = 12.59 and mean CpG methylation = 0.057), providing further validation that CpG island hyper-methylation silences RKIP expression. 

At the transcriptional level, direct binding of BTB domain and CNC homolog 1 (BACH1) and Snail1 (Snail) transcription factors to the RKIP promoter suppress RKIP transcription and expression [15,63]. Accordingly, the anticorrelated expression levels of RKIP, BACH1, and Snail are significant prognostic markers for metastasis-free survival of breast and prostate cancer patients [15,63]. RKIP promoter activity is also regulated by *cis*- and *trans*-acting elements within region −56 to +261 that responds to the transcription factors specificity protein 1 (Sp1), cyclic adenosine monophosphate (cAMP) response element-binding protein (CREB), and p300 [64]. Also, RKIP transcription is positively regulated via the direct binding of the androgen receptor (AR) to a putative androgen responsive element (ARE) within the RKIP promoter [65].

In various tumor models, RKIP mRNA has been shown to be targeted by miR-224 [66], miR-543 [67], miR-27a [68], and miR-23a [69], leading to its suppression. Contrarily, the long non-coding RNA (lncRNA) XIST stabilizes RKIP expression by suppressing miR-23a [70], while the lncRNA-GNAT-1 regulates the NF-κB/Snail cascade, an upstream inhibitor circuitry of RKIP expression [71]. The inhibitory NF-κB/YY1/Snail loop has also been reported to be targeted by forkhead box O4 (FOXO4), GATA binding factor, Sp1 and activation protein 4 (AP4), thus affecting indirectly RKIP expression [72,73].

At a post-translational level, a phosphorylation of RKIP at Serine 153 (pSer153-RKIP) by protein kinase C ζ (PKCζ) has been found in several cancer types to account for loss of RKIP activity as a Raf-1 proto-oncogene, serine/threonine kinase/mitogen-activated protein kinase kinase/extracellular signal-regulated kinase (Raf/MEK/ERK) and GPCR inhibitor, through inhibition of G protein-coupled receptor kinase 2(GRK2) [6,30,74,75,76,77,78]. In some cancers, the nuclear pSer153-RKIP levels significantly correlated with poor response to therapy and overall prognosis [75,79]. For example, RKIP overexpression in multiple myeloma mainly concerns its phosphorylated form (pSer153-RKIP), which impedes invasion and metastasis in preclinical animal models [36,80,81,82].

## 4. Central Signaling Pathways Regulated by RKIP

Under normal and pathophysiological conditions, including cancer, RKIP functions as a modulator of cellular growth, apoptosis, motility, genomic integrity, and therapeutic resistance [83]. RKIP critically interferes with the activation of basic upstream signaling pathways, which in turn govern the activity of individual downstream cascades essential for each of the above processes. Below, we focus on the central signaling cascades regulated by RKIP and discuss how their dysregulations in cancer confer to metastasis initiation and therapeutic resistance.

### 4.1. GPCR and MAPK Signaling Inhibition by RKIP

RKIP was first identified as an inhibitor of the Raf-1-stimulated MAPK signaling pathway (Raf-1/MEK/ERK) [3,8]. MAPK signaling is known to be hyperactivated in cancer cells and associated with metastasis initiation and therapeutic resistance [14,84]. RKIP functions as a competitive blocker of MEK phosphorylation by interrupting the Raf-1/MEK association, through direct binding to the Raf-1 kinase domain which prevents Ser338 and Tyr340/341 phosphorylation by PAK and Src Kinases, needed for Raf-1 activation [3,14,85]. Although RKIP may also associate with MEK and ERK, Raf-1 binding to RKIP and that of MEK are mutually exclusive [3,85]. Notably, B-Raf phosphorylation and activation are not directly affected by RKIP depletion, suggesting RKIP specificity for Raf-1 inhibition [7].

RKIP has been also reported to indirectly interfere with the activation status of upstream activators of Raf-1, such as GPCRs. The nature of this interference is mainly dictated by the phosphorylation status of RKIP. RKIP phosphorylation at Serine 153 (pS153) by PKC results in RKIP release from Raf-1 and binding to GRK2, an endogenous inhibitor of GPCR activation [6,86,87]. Binding of pS153/RKIP to GRK2 is causing the dissociation of GRK2 from GPCR, allowing GPCR activation and phosphorylation of downstream targets, including Raf-1. Skinner et al. demonstrated the presence of a phosphorylation-triggered salt-bridge competition, or “theft” mechanism, that controls the association between pSer153-RKIP and GRK2 [88]. Therefore, RKIP binding switch from Raf-1 to GRK2 bridges MAPK and GPCR signaling and implies its role as an endogenous modulator of cell response to growth factor stimuli.

### 4.2. IKK/IkBa/NF-κB Signaling Inhibition by RKIP

Constitutive activation of NF-κB is a hallmark not only in cancer initiation, progression, and metastasis, but also in tumor resistance to endogenous and exogenous apoptotic stimuli [89,90,91]. RKIP has been reported to negatively regulate NF-κB signaling, independently of MAPK, by antagonizing upstream signal transducers needed for NF-κB activation [1]. Yeung et al. first demonstrated physical interactions of RKIP with the upstream protein kinases of the NF-κB activating cascade, transforming growth factor beta-activated kinase 1 (TAK1) and NF-κB-inducing kinase (NIK), as well as with their targeted downstream kinases of the IkappaB kinase (IKK) complex, IKKα and IKKβ. The association of RKIP with NIK, TAK, IKKα, and IKKβ abolishes their kinase activity, which results in elimination of IkappaB α (IκBα) phosphorylation and degradation, leading therefore to inhibition of the NF-κB activity [1].

### 4.3. GSK3β Signaling by RKIP

The RKIP/GSK3β axis was first reported by Al Mulla and colleagues to have a critical impact in sustaining GSK3β activity, as a suppressor of multiple oncogenic pathways, including those of Wnt and cyclin D, known to be essential for tumor proliferation, progression, and metastasis [92]. RKIP can physically interact with GSK3β, preventing GSK3β phosphorylation at the inhibitory T390 residue by p38 MAPK, which is activated under oxidative stress augmented by RKIP depletion. RKIP dissociation from GSK3β de-represses GSK3β-mediated inhibition of cyclin D stabilization and induction of β-catenin, Snail and Slug, thus promoting tumor cell proliferation and EMT [92].

### 4.4. STAT3 Signaling Inhibition by RKIP

Constitutive activation of signal transducer and activator of transcription 3 (STAT3) in human tumors is associated, among others, with promotion of tumor cell EMT, migration, invasion, angiogenesis, and resistance to apoptosis [93,94,95,96]. RKIP blocks STAT3 activation, by preventing its phosphorylation by upstream kinases [97]. RKIP overexpression in breast and prostate cancer cell lines was shown to inhibit cellular Src (c-Src) autophosphorylation and activation, as well as interleukin 6 (IL-6)-, janus kinase 1/2 (JAK1/2)-, and activated Raf-mediated STAT3 tyrosine and serine phosphorylation, needed for STAT3 activation. Inhibition of c-Src- and JAK1/2-induced STAT3 tyrosine phosphorylation is mediated through a physical interaction of RKIP with STAT3 that blocks c-Src and STAT3 association. Contrarily, the inhibition of Raf-mediated STAT3 serine phosphorylation is attained through RKIP-induced suppression of Raf-1 activity [97]. Accordingly, RKIP expression has been found to be inversely correlated with phosphorylated STAT3 (pSTAT3) levels in different tumor types [75,76,98,99,100].

## 5. Major RKIP-Induced Metastasis Suppressor Signaling Modules

RKIP, as a master metastasis suppressor, exerts inhibitory functions at different steps of the metastatic process, including those that initiate metastasis such as angiogenesis, EMT, cell migration, and invasion [101,102]. RKIP targets signaling circuits that directly or indirectly regulate metastatic functions and which are mostly under the control of the central signaling pathways described above to be inhibited by RKIP. Therefore, the identification of RKIP acting sites throughout the metastatic process may reveal new targets for therapeutic intervention. Below, we present an updated overview of the cross-talks between RKIP and signaling modules involved in the regulation of the early metastatic events (Figure 2) (Table S1).

### 5.1. RKIP-Targeted MicroRNAs and Downstream Pro-Metastatic Factors

The microRNAs (miRNAs) have been suggested to play a vital role in almost all stages of tumor progression, through negative regulation of oncogenes and tumor suppressors [103]. One of the most well-characterized targets of RKIP is the let-7 family of microRNAs, which is known to suppress Ras-mediated MAPK activation and its subsequent effects on tumor invasion and metastasis [104]. Rosner and colleagues examined the underlying molecular mechanism of RKIP-induced let-7 upregulation in breast cancer metastasis models and showed that RKIP acts as a modulator of the Myc-Lin28-let-7 signaling cascade downstream of the Raf-1/MEK/ERK module [105,106]. RKIP, through MAPK inhibition, suppresses the activation of the ERK target Myc, which positively regulates the expression of let-7 inhibitor Lin28. As such, RKIP-mediated Myc inhibition downregulates Lin28 which in turn de-represses let-7, thus inhibiting tumor cell invasion and metastasis in vitro and in vivo [107,108].

Further studies in breast cancer models revealed that RKIP may also prevent tumor cell invasion and metastasis indirectly, by negative regulation of critical prometastatic factors downstream of let-7, including high mobility group AT-hook 2 (HMGA2) and BACH1 [106,109]. HMGA2 is a chromatin remodeling factor that promotes EMT and invasion by upregulating Snail, Slug, and Twist [14,18,110], while BACH1 is a transcription factor involved in bone metastasis of breast cancer by upregulating vital metastatic genes such as *chemokine receptor type 4* (*CXCR4*) and *matrix metalloproteinase 1* (*MMP-1*) [111]. Moreover, RKIP extends its indirect regulatory role downstream of HMGA2, in a number of additional let-7/HMGA2 targets involved in the metastatic process, as shown by gene and microRNA expression analyses performed in breast cancer cell lines [14]. The list of RKIP/let-7/HMGA2 targeted factors include, among others, syndecan (SDC2), miR-200b, lysine oxidase (LOX), miR-29, ten-eleven translocation 1 (TET1) and homeobox A-9 (HOXA9) [14,112]. In contrast to SDC2, miR-200b, LOX, and miR29 that positively regulate breast cancer initiation, EMT or invasion [113,114], the expression of the TET1 demethylase and its downstream target HOXA9 have been shown to suppress invasion, intravasation, extravasation, and metastasis of triple-negative breast cancer (TNBC) cell lines in vitro and in vivo [14,112]. HMGA2 inhibition after ectopic RKIP induction in breast cancer cell lines resulted in induction of miR200b, which in turn downregulated its direct target LOX, thus leading to decreased tumor cell invasion and metastasis [109]. RKIP-mediated HMGA2 downregulation also directly inhibited the expression of the pro-metastatic factor SDC2 independently of miR-200b, resulting in suppression of tumor survival and metastasis [109]. Furthermore, HMGA2 depletion in breast cancer cells resulted in downregulation of miR-29 and induction of its target TET1, which in turn demethylates the promoter of the metastasis-suppressor HOXA9 and promotes its transcription [112]. RKIP has been further shown to potentiate HMGA2 inhibition and metastasis suppression independently of the Lin28/let-7 axis. RKIP overexpression in glioma cell lines was found to increase the expression of miR-98, which in turn inhibits its target gene *HMGA2*, resulting in decreased glioma cell invasion but without affecting tumor cell proliferation rates [115]. Concomitantly, in breast cancer models, RKIP induction was able to suppress tumor cell proliferation and invasion by upregulating miR-185, an upstream negative regulator of HMGA2 [116].

### 5.2. RKIP-Mediated NF-κB/YY1/Snail Circuity Inhibition

NF-κB signaling plays a critical role in tumor angiogenesis and the early metastatic events such as EMT. The list of NF-κB-targeted genes involved in angiogenesis include basic fibroblast growth factor (*b*-*FGF*), *IL*-*8*, *MMP*-*2*, -*3*, and -*9*, while NF-κB inhibition abolishes vascular endothelial growth factor (VEGF) expression and suppresses angiogenesis [89,117,118]. NF-κB promotes EMT, cell migration and invasion not only by inducing the expression of MMPs, but also by regulating directly or indirectly the transcription of key EMT modulators, including the transcription factors Snail and Twist, as well as of cell adhesion molecules, such as selectins, integrins, and their ligands [119,120,121,122]. We and others have reported the role of drug-induced or ectopic RKIP expression in various cancer cell lines in the modulation of NF-κB signaling and its downstream targets that confer to the EMT phenotype and migratory and invasive properties of cancer cells [123,124,125,126,127,128]. For example, overexpression of RKIP in breast and pancreatic adenocarcinomas was able to suppress NF-κB-dependent tumor cell invasion through downregulation of MMPs [124,125]. Notably in melanoma RKIP was found to inhibit melanoma differentiation antigen-9 (MDA-9)/syntenin-mediated tumor migration and metastasis through physical association with MDA-9, that disturbs the assembly of stable c-Src/focal adhesion kinase (FAK) signaling complexes, which is essential for the activation of NF-κB and MMPs [129]. Treatment of prostate cancer cell lines with the proteasome inhibitor NPI-0052 or the nitric oxide donor DETA/NO was shown, in vitro and in vivo, to reverse tumor cell EMT, migration, and invasion through, at least, RKIP-mediated NF-κB signaling inhibition and consequent suppression of the EMT inducers, Snail, vimentin, N-catherin, and fibronectin, and E-cadherin upregulation [130,131]. RKIP, therefore, may participate in the indirect regulation of its transcriptional suppressor, Snail, via NF-κB inhibition [15,130,131,132,133]. Our preliminary findings have further identified an additional oncogene and NF-κB target, Yin Yang 1 (YY1), to play a vital role in prostate cancer metastasis, since YY1 silencing in metastatic prostate cancer cell lines was able to inhibit TGFβ-induced EMT. YY1 involvement in regulating breast cancer metastasis has been previously reported [134]. RKIP overexpression inhibited YY1 mRNA and protein expressions, most likely via NF-κB inhibition [135]. Since Snail transcription is directly activated by YY1 [136], we can hypothesize that RKIP eliminates tumor EMT characteristics by acting as a negative regulator of the NF-κB/YY1/Snail circuit [137]. These findings highlight the significance of NF-κB/YY1/Snail circuitry targeting in cancer by various molecules with RKIP-inducing activities for reversal of EMT transformation [74,123,138,139]. Last, but not least, RKIP upregulation after Snail knockdown has also been shown to reduce the expression of colon cancer stem cell (CSC) markers leading to reversal of EMT [140].

### 5.3. RKIP-Targeted STAT3 Inhibition

Cytokine/growth factor-activated Src and STAT3 have been linked with promotion of EMT, migration, invasion, and angiogenesis in cancer cells [93,94,95,97,141,142], through induction of STAT3 downstream metastasis-associated proteins, including mucin 1 (Muc1), VEGF, and CXCR4, and by microtubule stabilization [143]. RKIP overexpression was reported to reverse STAT3-dependent prostate and breast cancer invasive and migratory properties, through a direct constitutive physical interaction with STAT3 that blocks c-Src and STAT3 association, needed for STAT3 activation [97]. These findings demonstrate that RKIP negates tumor angiogenesis, EMT and migration by interrupting the expression of Muc1, CXCR4, and VEGF [97]. A different study has indicated a physical interaction between RKIP and Annexin A7 (ANXA7), a suppressor of tumorigenesis and metastasis in prostate cancer. This interaction impairs the activation of ANXA7 GTPase by a small molecule SEC, which in turn leads to metastasis suppression via the AMPK/mammalian target of rapamycin complex 1 (mTORC1)/STAT3 signaling pathway [144]. The inhibitory role of RKIP on STAT3 signaling and the consequent effects on metastasis initiation in vitro and in vivo have been also validated by additional studies on other tumor models [98,99,145].

### 5.4. RKIP-Targeted Suppression of MMPs

The overexpression of multiple MMPs is a hallmark in tumor invasion and metastasis [146]. In many cancers, the expression of MMPs such as -1, -3, -10, and -13 is negatively correlated with RKIP levels, suggesting that RKIP may constrain metastasis through inhibition of MMPs [147]. Interestingly, it has been proposed that the expression signature of RKIP and MMPs is better at predicting high metastatic risks in various cancers than the individual protein [102]. MMPs expression is induced by Raf/MEK/ERK, IKK/NF-κB, AKT serine/threonine kinase 1 (Akt) and STAT3 cascades, known to be inhibited by RKIP [93,148,149,150]. Along with the MMP1 inhibition by the RKIP/let-7/BACH1 axis in breast cancer cells, RKIP may exert inhibitory activities on MMPs expression through alternative signaling axes. For example, RKIP has been reported to inhibit local breast cancer invasion by antagonizing the transcriptional activation of MMP-13, mediated by the ERK2 signaling pathway and independently of the ERK2 downstream target activator protein 1 (AP-1) [102]. In contrast, MMP1 activation in the same tumor model was found to be RKIP- and AP-1-dependent. Erk2 but not Erk1 is known to induce EMT transformation in breast cancer cells [151]; however, the role of RKIP in inhibiting breast cancer cell EMT is still under investigation. Furthermore, the inhibition of glioma cell invasion and migration through RKIP-mediated suppression of MMP-2 and MMP-9, along with HMGA2, has also been reported by Lei et al. [152].

### 5.5. Other Critical Interactions of RKIP with Metastasis Regulators

Recent studies have provided evidence that RKIP is likely an upstream regulator of the Notch1 pathway in cancer cells [153]. The Notch pathway has been found aberrantly activated in many solid tumors, and its activation is functionally associated with different stages of tumor metastasis, including EMT [154,155,156]. Upon activation by ligand binding, Notch1 releases its intracellular domain (NICD), which subsequently translocate into the nucleus, where together with other transcription factors forms a transcriptional complex, responsible for the activation of EMT inducing genes, such as *Slug* and *Snail* [155,156,157,158]. Noh et al. showed that RKIP activity and levels in cervical and stomach cancer cell lines and tissues are inversely correlated with endogenous levels of NICD and that RKIP overexpression resulted in significant NICD reduction with subsequent inhibition of NICD-mediated expression of EMT markers, including vimentin, N-cadherin, and Snail. It was proposed that RKIP may attenuate Notch1 signaling through direct physical interaction; however, this interaction cannot affect the proteolytic release of NICD needed for Notch1 activation, thus suggesting that RKIP may play a distinct role in Notch1 activation during EMT and metastasis [153].

### 5.6. RKIP-Targeted Tumor Microenvironment Components

Recent findings suggest a strong involvement of metastasis regulators in modulating components of the tumor microenvironment and vice versa [159,160]. Besides cancer cells, the tumor microenvironment consists of different cell populations with pleiotropic functions in controlling tumor progression and metastasis. Among them, infiltrating immune cells, such as tumor-associated macrophages (TAMs) and myeloid cells, are known to add to the invasive phenotype of tumor cells [161].

In 2015, Rosner’s and Yeung’s groups reported, almost simultaneously, the role of RKIP in controlling macrophage infiltration in the breast cancer microenvironments, in vitro and in vivo [101,160]. RKIP expression in triple-negative breast tumors significantly reduced the number and metastatic potential of infiltrating TAMs. The underlying mechanism of TAM recruitment inhibition involved RKIP-mediated HMGA2 blockage which, in turn, induced a reduced expression of macrophage chemotactic factors, such as chemokine ligand 5 (CCL5), by cancer cells. The incompetence of the tumor microenvironment to be enriched by TAMs due to RKIP-mediated CCL5 reduction was translated into a decreased tumor cell invasiveness and secretion of pro-metastatic factors, including tumor necrosis factor receptor 2 (TNFR2) and progranulin (PRGN), by TAMs [160]. In conjunction with Frankenberger et al. findings, Datar et al. also reported that the inhibition of CCL5 by ectopic RKIP expression in an orthotopic breast cancer model was the cause of significant reductions observed in tumor vascularization, macrophage infiltration, and lung metastases [101]. Accordingly, the latter study established for the first time an inverse correlation between RKIP and CCL5 expression levels in clinical human breast cancer samples. Altogether, both studies identified RKIP as a novel negative regulator of the tumor microenvironment, at least by blocking the recruitment of pro-metastatic macrophages, through vital chemokine regulation.

Furthermore, RKIP expression was positively correlated with gene signatures involved in effective T cell responses during immunotherapy of metastatic melanoma with dendritic cell (DC) vaccination, but inversely correlated with genes associated with myeloid cell infiltration and inflammation such as STAT3, Notch1, and MAPK1 signaling members [162]. Similarly, in gastric cardiac adenocarcinoma tissues, negative RKIP expression coincided with lower T cell-mediated immune function in the tumor microenvironment and increased lymph node metastasis, possibly through NF-κB hyperactivity [163].

Summarizing, there is accumulating evidence supportive for the involvement of RKIP in the regulation of the tumor microenvironment, as it relates to the potency of specific immune cell infiltration and production of pro-metastatic factors.

## 6. RKIP as a Chemo-Immuno-Radio-Therapeutic Sensitizer in Cancer

Cancer cells often develop therapeutic resistance along with increased metastatic potential, thus rendering the treatment less effective and the overall disease prognosis quite poor. In many cases, therapeutic resistance also coexists with increased likelihood of tumor cell escape host-immuno-surveillance, such as immuno-mediated cytotoxicity. These coincidences may be attributed to dysfunction of signaling pathways that control tumor responses to both apoptotic and metastatic stimuli. Therefore, understanding the common underlying molecular mechanisms and identifying gene products able to simultaneously regulate the involved signaling cascades in cancer cells are important steps in addressing therapeutic resistance and metastasis management options in human malignancies.

**Figure 3:**
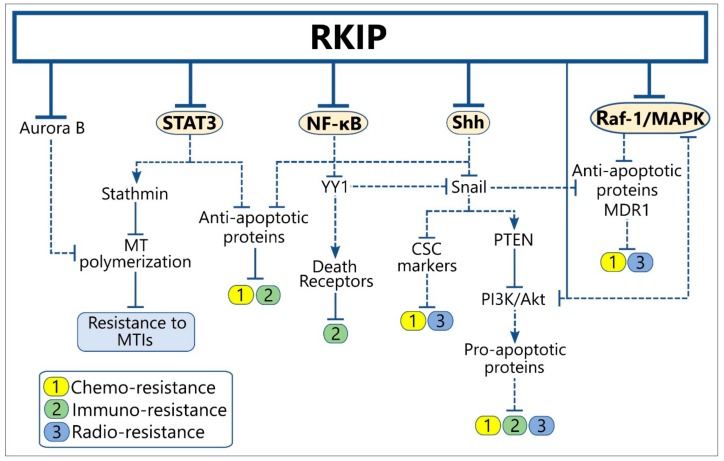
RKIP cross-talks with signaling modules regulating tumor resistance to therapy and host immune-surveillance. Solid lines indicate the physiological function of each protein on the expression of downstream targets, while the dotted lines show the downstream effect(s) of the indicated protein after alteration of its levels by RKIP. Abbreviations: CSCs, Cancer Stem Cells; MDR, Multi-Drug Resistance; MT, Microtubule; MTIs, Microtubule Inhibitors.

We and others have implicated RKIP expression in regulating, along with the metastatic process, the tumor cell resistance to conventional chemotherapy, radiotherapy, and immuno-mediated cytotoxicity [29,164]. In the cancer models studied so far, RKIP functions as an apoptosis inducer, causing re-sensitization of resistant tumors to conventional therapeutic modalities and/or sensitivity to host immuno-surveillance. This is achieved via multiple interactions with signaling modules whose activation is known to confer to the resistant cancer cell phenotype (Figure 3) (Table S2). Interestingly, many of the implicated RKIP-modulated cascades are also involved in metastasis regulation, thus suggesting that RKIP may exert a dual function in metastasis and resistance by affecting common regulatory paths. Below, we summarize the most reported mechanisms of RKIP-mediated reversal of tumor therapeutic resistance.

### 6.1. RKIP-Mediated Inhibition of NF-κB and Snail Signaling

NF-κB activity promotes tumor resistance to conventional chemotherapy and immuno-mediated cytotoxicity mainly by inducing the expression of B-cell lymphoma 2 (Bcl-2)-related anti-apoptotic gene products, cellular FLICE (FADD-like IL-1β-converting enzyme)-inhibitory protein (c-FLIP) and inhibitor of apoptosis proteins 1/2 c-IAP1/2 and X-linked inhibitor of apoptosis protein (XIAP), as well as by regulating death receptor expression [90,91]. We and others have reported that small molecules such as NPI-0052 and DETA/NO, or immunomodulating agents including αnti-CD20 and αnti-CD80 antibodies, are able to sensitize various resistant cancer cell lines to both chemo- and TNF-related apoptosis-inducing ligand (TRAIL)-mediated apoptosis, through NF-ΚB and Snail signaling inhibition and RKIP induction [26,74,123,126,138,165,166]. NF-κB constitutive activity has been also associated with adaptive tumor resistance to ionizing radiation [167]. The direct suppressive effects of RKIP ectopic induction or Snail silencing in the expression of the downstream anti-apoptotic proteins of the Bcl-2 family, as well as in the type II apoptotic pathway activation, support the opposing roles of RKIP and the NF-κB/Snail module in the regulation of tumor chemo/immuno-resistance [26,126].

Furthermore, drug-induced Snail overexpression in colon cancer cell lines has been shown to corelate with elevated expression of CSC markers, such as CD133, Nanog, and Oct4, and increased chemoresistance. The above events were able to be reversed by Snail silencing and RKIP upregulation, suggesting that the Snail/RKIP loop is a critical regulatory component of CSC existence within the tumor, which in turn is associated with tumor chemoresistance [140]. Alongside, RKIP reduction enhanced non-small-cell lung cancer (NSCLC) radio-resistance via sonic hedgehog (Shh) signaling activation [168], which is known to accelerate CSC marker expression and sustain CSC self-renewal and functional properties partially through induction of Snail expression [169,170]. Altogether, the above findings demonstrate RKIP as a negative regulator of radio- and chemo-resistance by affecting the number and functions of CSCs within the tumor.

YY1 has been implicated, by us and others, in promoting therapeutic resistance and immunoescape in solid and hematological malignancies [22,171,172,173,174]. We have accumulating evidence demonstrating that YY1, as a downstream target of NF-κB, can be modulated by RKIP-mediated NF-κB inhibition, thus abolishing its activity on cancer cell resistance [22,30,135]. RKIP-induced YY1 inhibition may further contribute to Snail suppression, given that YY1 acts as a direct transcriptional activator of Snail [30,74,136]. YY1, therefore, may function as a linker between NF-κB and Snail activation statuses, that will critically affect the activity of downstream cell death apoptotic pathways [22,123]. Consequently, the modulatory ability of RKIP towards the NF-κB/YY1/Snail circuitry can be considered as one of the underlying mechanisms of RKIP-mediated inhibition of tumor chemo/immuno-resistance [22,30]. Accordingly, agents able to induce RKIP expression or to mimic RKIP function in promoting NF-κB/YY1/Snail suppression are of therapeutic importance for the reversal of acquired therapeutic resistance, including photo-resistance [26,74,123,138,172,175,176,177].

### 6.2. RKIP-Mediated Death Receptor Induction

There is strong evidence for RKIP implication in the indirect regulation of death receptors (DR) expression, by inhibiting their transcriptional repression and, therefore, increasing cell sensitivity to immuno-mediated cytotoxicity. An underlying mechanism of DR induction by RKIP in cancer cells is through inhibition of the NF-κB/YY1 cascade. We and others have shown that YY1 acts as transcriptional repressor of DR5 and Fas receptors in different cell types [173,178]. The de-repression of death receptor transcription by RKIP-promoting drugs (NPI-0052, DETA/NO, etc.), or RKIP overexpression abrogates tumor resistance to TRAIL and Fas-ligand (FasL)-mediated apoptosis [26,31,34,74,80,139,179,180,181,182,183,184,185]. Indeed, YY1 levels have been found inversely correlated with DR5, Fas, and RKIP expressions in multiple cancer tissues [30,73,186,187].

We have also shown that the sensitizing activity of immune-modulatory agents, such as the αnti-CD20 monoclonal antibodies (mAbs) Rituximab, LFB-603, and BM-ca, on B-cell Non-Hodgkins lymphoma (B-NHL) cell lines resistant to TRAIL/FasL- and natural killer (NK)-induced apoptosis also involved Ab-mediated YY1 inhibition [74,183,188,189]. Given that all the above agents induced RKIP expression, and RKIP overexpression suppresses YY1, we can suggest that the tested agents enhance their sensitizing action to apoptotic death, through RKIP-mediated YY1 inhibition and death receptor upregulation. In addition, we cannot exclude, although never tested so far, that RKIP induction by αnti-CD20 Abs may further interfere with the potency of antibody-dependent cell-mediated cytotoxicity (ADCC) and complement dependent cytotoxicity (CDC), known to be also mediated by therapeutic αnti-CD20 Abs.

### 6.3. RKIP-Mediated MAPK and PI3K/AKT Signaling Inhibition

The MAPK signaling dynamics influences tumor response to conventional chemotherapy, as demonstrated by a number of studies in multiple preclinical models of solid and hematological malignancies [24]. The underlying molecular mechanisms of resistance involve profound effects of the Raf/MEK/ERK pathway on the expression and activities of drug pumps and apoptosis-related proteins such as the multi-drug resistant-1 (MDR-1), Bad, Bim, Mcl-1, caspase 9, and Bcl-2, resulting in inhibition of drug-induced apoptosis [190,191,192,193,194,195]. These effects mainly occur at the transcriptional and post-translational levels, as the activation of Raf/MEK/ERK may induce the phosphorylation of transcription factors that bind to the promoters of MDR-1 and Bcl-2, thus stimulating their transcription. Raf/MEK/ERK activation may also promote the phosphorylation of the anti-apoptotic Mcl-1 protein and the pro-apoptotic Bim protein, resulting in the activation and proteasome degradation of the respective proteins [196,197,198]. Similarly, constitutive activation of the PI3K/Akt signaling in cancer cells has been also associated with apoptosis reduction, via several mechanisms [199].

In some cancer types, the MAPK- and/or PI3K/Akt-dependent radio-resistance and/or chemo-resistance has been reported to be promoted by RKIP reduction, via constitutive phosphorylation and activation of ERK and/or Akt [34,200,201,202]. Along with NF-κB, downstream targets of both ERK and PI3K/Akt signaling are known to be implicated in the negative regulation of apoptosis induced by conventional chemotherapy or ionizing radiation [201,203,204,205,206,207]. Given that both pathways are activated by GPCR/Ras signaling and cross-talking at multiple levels [208,209,210,211,212,213], it is expected that the inhibitory function of RKIP on GPCR activation and Ras/Raf/MEK/ERK signaling may also affect Akt activity. Indeed, ectopic RKIP expression or upregulation by chemo/immune-modulatory agents, increased tumor chemo- and radio-sensitivity in vitro, by suppressing MAPK and/or PI3K activation [34,200,201].

Another proposed mechanism of Akt signaling inhibition is through RKIP-mediated modulation of the NF-κB/YY1/Snail/ phosphatase and tensin homolog (PTEN) circuitry, which can act independently of MAPK inhibition [175]. We have shown that RKIP induction in NHL cell lines by the LF-603 αnti-CD20 mAb co-exists with upregulation of the tumor suppressor phosphatase and tensin homologue (PTEN) and Snail/YY1 inhibition, resulting in tumor immune-sensitization [74]. As PTEN is an endogenous inhibitor of the PI3K/Akt signaling [214] and it is transcriptionally repressed by Snail [215], we suggest that RKIP-mediated NF-κB/YY1/Snail inhibition may trigger Akt inactivation by PTEN induction, leading to subsequent reversal of the resistant tumor phenotype.

### 6.4. RKIP-Mediated Reversal of Tumor Resistance by STAT3 Inhibition

There is substantial evidence demonstrating RKIP as a critical player in opposing the effects of pro-oncogenic STAT3 activation in cell survival and therapeutic resistance. Constitutive STAT3 activation in cancer cells has been associated with enhanced transcription of anti-apoptotic genes, thus increasing tumor resistance to apoptosis and promoting neoplastic progression [216,217]. RKIP overexpression has been reported by Yousuf et al. to reverse breast and prostate cancer resistance to microtubule inhibitors (MTIs) by inhibiting STAT3 activity in vitro and in vivo [97]. As an underlying mechanism, it was proposed the interruption of c-Src-STAT3 association by RKIP, that leads to insufficient STAT3-mediated inhibition of the microtubule-destabilizing protein Stathmin, which is needed for microtubule polymerization [143]. These findings can be explained by the recently reported role of RKIP in mediating microtubule dynamics in cancer cells by regulating mitotic spindle checkpoints via association with centrosomes and kinetochores through Aurora B kinase [4]. 

The significance of RKIP-mediated STAT3 regulation on tumor survival has been also highlighted by other studies. IL-6-mediated activation of STAT3 in colon cancer cell lines was shown to occur in conjunction with the phosphorylation (inactivation) of RKIP, leading to poor prognosis of colon cancer stage II patients. Cell treatment with oxaliplatin and camptothecin was able to block IL-6-mediated STAT3 activation and RKIP phosphorylation via the inhibition of STAT3/gp130 interaction [75].

## 7. RKIP as a Prognostic Indicator and a Therapeutic Target

### 7.1. RKIP’s Prognostic Value

The involvement of RKIP in cancer progression and metastasis was suggested almost a decade ago [15]. Since then, several studies have evaluated RKIP as a prognostic factor for survival in different cancers and emerging evidence suggests its use as a therapeutic target [9,19,218,219]. The loss of RKIP is an independent indicator of poor prognosis in patients with digestive tract cancers, including esophageal, gastric, and colorectal cancer [220]. Low RKIP expression or its loss associates with the onset and development of gastric cancers and its ability to invade and metastasize [57,221], as well as with recurrence in esophageal squamous cell carcinoma (ESCC) [222], malignant progression in hepatic fibrosis [223], and poor survival in gastric cancer [224,225]. RKIP loss also serves as a predictive marker for the progression and metastasis of liver [226,227], kidney [228], breast [229], ovarian [230], and colorectal cancers [231], as well as pancreatic ductal adenocarcinoma (PDAC) [232]. Combined with its promoter’s methylation, RKIP expression was further suggested as a biomarker of ESCC [233]. Phospho-RKIP was also reported as a predictive indicator of survival in lung cancer [234].

Furthermore, RKIP expression correlated negatively with disease-specific survival [235] in patients with colorectal and ductal breast cancers [19,236]. Furthermore, along with the expression of urokinase plasminogen activator receptor (uPAR), the proliferative index and tumor border configuration, RKIP expression was identified as a decisive classifier for the identification of colorectal tumors with vascular invasion [237,238,239]. Noteworthy, Shvartsur et al. constructed various possible cross-talks between RKIP (active/inactive) and the gene products underlying the mechanism of RKIP overexpression in multiple myeloma, aiming to use such molecular signatures for a more precise diagnosis/prognosis of the disease [80]. 

### 7.2. RKIP-Inducing Agents 

Since RKIP is commonly downregulated in the majority of the human cancers, several drugs could potentially be used to upregulate its expression. These can be either synthetic drugs or natural agents, but treatment options also include specific proteins and microRNAs, as mentioned above [11].

The synthetic agents/drugs being used induce the interaction of RKIP with its partners in the MAPK and PKC pathways, and thus inhibit tumor progression, metastasis, and EMT [83]. Such drugs include rituximab [240], dihydroartemisinin [241], and didymin [242], all of which upregulate RKIP protein expression. Moreover, Shogaol, a constituent of ginger similar in chemical structure to gingerol, has been used to increase RKIP levels [243], as well as camptothecin and oxaloplatin, both of which inhibit RKIP phosphorylation [75]. Furthermore, we have shown that nitric oxide (NO) donors, such as DETA/NO, upregulate RKIP by inhibiting the NF-κB/YY1/Snail regulatory circuitry resulting in tumor chemo-immuno-sensitization and inhibition of EMT and metastasis [31,123,175]. The potential therapeutic use of NO donors was also suggested in the treatment of patients with refractory cancers and in the prevention of the initiation of the metastatic cascade via EMT [123].

More recently, the regulation of RKIP using different natural agents is attracting interest. Its expression was significantly boosted in PDAC cells that were treated with epigallocatechin gallate (EGCG). EGCG management also impeded both the nuclear accumulation of NF-κB and functionally active ERK [125]. Furthermore, ginseng extract (*Panaxquinquefolius* L.) could reduce phospho-ERK1/2 and -MEK1/2 levels and increase RKIP and pRaf-1 in breast carcinoma cells [244]. NPI-0052 is a natural proteasome inhibitor shown by us and others to induce RKIP via NF-κB/Snail inhibition, resulting in reversal of prostate cancer cell EMT, migration, invasion, and chemo/immune sensitization [26,130]. Apart from inhibiting NF-κB, proteasome inhibitors restore RKIP levels by impeding proteasome degradation of the ubiquitinated protein in triple-negative breast cancer cells [245].

## 8. Concluding Remarks

It is clear that the pleiotropic activities of RKIP in cancer have evolved rapidly since its discovery in 1999. RKIP manifests inhibitory functions in central signaling pathways involved in the promotion of most tumor-related properties. RKIP low/absent expression in the majority of cancers underlies, among others, tumor response to apoptotic and metastatic stimuli. Suppression of tumor cell therapeutic resistance, angiogenesis and metastasis initiation and progression by RKIP emphasizes the potential of high RKIP levels in re-sensitizing resistant tumors and reducing the risk of aggressive tumors to metastasize. Therefore, it may be crucial to identify available modalities with RKIP-promoting activities, or develop new agents targeting RKIP overexpression, that can be used alone or in combination, to abolish the cancer-related properties listed above and resulting in novel successful therapeutic approaches for all cancers.

## Supplementary Materials

The following are available online at http://www.mdpi.com/2072-6694/10/9/287/s1, Table S1: Reported signaling modules whose modification by RKIP contributes to inhibition of metastasis initiation in various cancer types, Table S2: Reported signaling modules whose modification by RKIP contributes to inhibition of therapeutic resistance in various cancer types.
