# Supplementary material for: RKIP: A Key Regulator in Tumor Metastasis Initiation and Resistance to Apoptosis: Therapeutic Targeting and Impact
Source: Cancers (Basel). 2018 Aug 24;10(9):287. doi: 10.3390/cancers10090287 (PMC6162400; doi:10.3390/cancers10090287)
Supplement: Supplementary file 1 [file cancers-10-00287-s001.pdf]

Supplementary Materials: RKIP: A Key Regulator in Tumor Metastasis Initiation and Resistance to Apoptosis: Therapeutic Targeting and Impact

Apostolos Zaravinos, Benjamin Bonavida, Ekaterini Chatzaki and Stavroula Baritaki

Table S1. Reported signaling modules whose modification by RKIP contributes to inhibition of metastasis initiation in various cancer types. PF, Preliminary Findings; CSC, Cancer Stem Cells; PDAC, Pancreatic Adenocarcinoma; GCA, Gastric Cardia Adenocarcinoma; HCC, Hepatocellular Carcinoma; CRC, Colorectal Adenocarcinoma; NSCLL, Non-Small Cell Lung Cancer; LN, Lymph Node; NPC, Nasopharyngeal Carcinoma; IDC, Invasive Ductal Carcinoma; TNBC, Triple Negative Breast Cancer; TAM, Tumor Associated Macrophages; OVCA, Ovarian Carcinoma; LAC, Lung Adenocarcinoma.

**Signaling Module**	**Cancer Type**	**Mechanism**	**Effect**	**References**
MAPK/Myc/Lin28/let-7	Breast	↓ Let-7 targets	↓ Invasion<br>Intravasation<br>Bone metastasis	[18,105–107]
Lin28 (MAPK-independent)	Esophageal	GRK-2, MMP-14	Invasion<br>LN metastasis	[147]
MAPK/let-7/HMGA2	Breast	↓ CDC2, LOX, OPN, miR-29, miR-200,<br>Snail, Slug, Twist,	↓ EMT, invasion	[14,18,106,110,<br>113,114,109]
	Breast	↑ TET1, HOXA	Invasion/Intravasation	[14,112]
Let-7/BACH1	Breast	↓ CXCR4, MMP-1	Extravasation<br>Bone metastasis	[63,106,111]
<u>*Let-7 independent modules*</u>				
MAPK/HMGA2	Glioma	MMP-2, MMP-9	Invasion, Migration	[152]
	TNBC	TAM recruitment<br>CCL5, TNFR2, PRGN	Invasion, Intravasation	[101,160]
miR-98/HMGA2	Glioma	HMGA2 targets	Invasion	[115]

miR-185/HMGA2	Breast	HMGA2 targets	Invasion	[116]
Raf-1/MEK/ERK1/2	PDAC	↓ ERK1/2 targets	↓ Migration, invasion	[84]
	NPC		Invasion	[46]
	CRC			[19,21,202]
	HCC			[127,226]
	OVCA			[230]
Raf-1/MEK/ERK2	Breast	MMP-13	Invasion	[102]
Raf-1MEK/ERK2/AP-1	Breast	MMP-1	Invasion	[151]
	Melanoma	AP1-targets	Invasion	[58]
NF-κB	Breast	↓ MMPs	↓ Invasion	[19,125]
	CRC			
	Melanoma			
	HCC		Invasion, Migration	[127]
	Melanoma	MDA-9/ c-Src/FAK	Invasion, Metastasis	[129]
	GCA	↑ T-cell response	LN metastasis	[163]
NF-κB/Snail,Twist	Prostate	↓ EMT markers	↓ EMT	
	Breast	Epithelial markers	Migration	[15,52,62,120,123,
			Invasion	128,130,131,243]
	CRC	↓ CSC markers	EMT	[21,140]
		EMT markers	Invasion, Migration	
NF-kB/YY1/Snail	Prostate	↓ EMT	↓ EMT	PF, [134–138]
	Breast	Invasion markers	Invasion	
c-Src /STAT3	Prostate	↓ Muc1, CXCR4, VEGF	↓ Angiogenesis	[97]
	Breast		Migration, Invasion	
	Gastric	?	LN metastasis	[98]

JAK/STAT3<br><br>AMPK/mTORC1/STAT3	NSCLC<br>NPC<br>Prostate	?<br>EMT-markers<br>ANXA7/ ANXA7 GTPase	Invasion<br>EMT/Invasion/Migration<br>Metastasis	[99]<br>[145]<br>[144]
Notc1/NICD	Cervical<br>Stomach	↓ Snail, EMT markers	↓ EMT	[153–156]
GSK3β/Snail	CRC	↓ β-catenin, Snail, Slug	↓ EMT	[92]
PI3K/Akt (with or without PTEN involvement)	Melanoma<br>Prostate<br>Neuroblastoma	↓ Snail, YY1, MMPs<br><br>CD31, N-myc, vimentin	↓ Migration<br>Invasion<br>Angiogenesis	PF, [40]<br>PF, [137]<br>[242]
Unidentified modules	Gastric<br>IDC<br>TNBC<br>Prostate<br>PDAC<br>LAC	↓ MMP-1, MMP-2<br><br>miR-27a	↓ Angiogenesis<br>Invasion<br>Migration<br>Vascular Invasion<br>EMT<br>EMT	[57]<br>[35]<br>[10]<br>[17]<br>[232]<br>[68]

Table S2. Reported signaling modules whose modification by RKIP contributes to inhibition of therapeutic resistance in various cancer types. PF, Preliminary Findings; IS, Immunosurveillance; IT, Immunotherapy; MTIs, Microtubule Inhibitors; PDT, Photodynamic Therapy; CSC, Cancer Stem Cells; MDR, Multi-Drug Resistance; B-NHL, B-cell Non-Hodgkins Lymphoma; HCC, Hepatocellular Carcinoma; CRC, Colorectal Adenocarcinoma; NPC, Nasopharyngeal Carcinoma; LAC, Lung Adenocarcinoma; GCA, Gastric Cardia Adenocarcinoma; NSCLL, Non-Small Cell Lung Cancer.

**Signaling Module**	**Cancer Type**	**Mechanism**	**Effect**	**Ref.**
NF-κB (direct effect)	Prostate<br>Breast<br>Prostate<br><br>GCA	↑ PARP, caspase 8, FLIP<br><br>↓ anti-apoptotic proteins<br>↑ pro-apoptotic proteins<br>T-cell responses	↓ Chemo-resistance<br><br>Chemo-resistance<br>TRAIL-resistance<br>Resistance to host IS	[27]<br><br>[22,34,123,<br>124,138]<br>[163]

NF-kB/Snail<br><br><br><br><br>NF-κB/ΥΥ1<br><br><br><br>NF-κB…/PTEN	Prostate<br><br><br>CRC<br><br><br>B-NHL<br>prostate<br><br><br>B-NHL	↓ Bcl-xL, XIAP, cyt-C,<br>↑ caspases-8 /9,<br><br>↓ CSC markers, MDR1, ABCG2, anti-apoptotic proteins<br><br><br>↓ Snail, Bcl-2/Bcl-xL, Mcl-1<br>↑ DR5, Fas<br><br><br>↓ Snail, pAkt	↓ Chemo-resistance<br>TRAIL-resistance<br>Resistance to PDT<br>TRAIL resistance<br>Chemo-resistance<br><br>↓ Chemo-resistance<br>TRAIL/FasL-resistance<br><br><br>TRAIL-resistance<br>Chemoresistance	[22,34,123,124<br>165,29]<br>[177]<br>[140]<br><br><br>[22,28–30,33,34,124,138,139,172,173,183,184,189]<br>[74,215]
Raf-1/MEK/ERK1/2	Prostate<br>Breast<br>HCC<br>CRC<br>NPC<br>B-NHL<br>Melanoma	↓ AP-1 anti-apoptotic targets<br><br>ERK-anti-apoptotic targets<br><br><br>Bcl-xL<br>↑ T-cell response genes	↓ Chemo-resistance<br><br>Chemoresistance<br>Chemo-resistance<br>Radio-resistance<br>Chemo-resistance<br>Resistance to DC IT	[27]<br><br>[58,200]<br>[202]<br>[164,201]<br>[32]<br>[162]
PI3K/Akt	NPC<br>neuroblastoma	↓ Akt-anti-apoptotic targets<br>Bcl-2, cyclin D1, CDK4	↓ Radio-resistance<br>Resistance to apoptosis	[201]<br>[242]
Shh/Gli1	NSCLC	↓ Snail<br>CSC markers and function	↓ Radio-resistance	[168,169]
c-Src/IL-6/ or Jak1/2-activated STAT3	Breast<br>Prostate	↑ Stathmin<br>↓ MT stabilization	↓ Resistance to MTIs	[4,97]
Unidentified modules	LAC	↓ miR-27a anti-apoptotic targets	↓ Chemo-resistance	[68]

	Cervical	Bcl-2	↓ Resistance to apoptosis	[241]
